# Combination of PIM and JAK2 inhibitors synergistically suppresses cell proliferation and overcomes drug resistance of myeloproliferative neoplasms

**DOI:** 10.18632/oncotarget.1951

**Published:** 2014-05-08

**Authors:** Shih-Min A. Huang, Anlai Wang, Rita Greco, Zhifang Li, Fangxian Sun, Claude Barberis, Michel Tabart, Vinod Patel, Laurent Schio, Raelene Hurley, Bo Chen, Hong Cheng, Christoph Lengauer, Jack Pollard, James Watters, Carlos Garcia-Echeverria, Dmitri Wiederschain, Francisco Adrian, JingXin Zhang

**Affiliations:** ^1^ Sanofi Oncology, Cambridge, MA, 02139, USA; ^2^ Current affiliation: Genentech, 1 DNA way, South San Francisco, CA, 94080, USA; ^3^ Sanofi, Lead Generation and Candidate Realization, Waltham, MA USA; ^4^ Sanofi Oncology, Discovery and Early Development, Vitry-Sur-Seine, France; ^5^ Sanofi- Genzyme, GDB, Cambridge, MA, 02139, USA; ^6^ Current affiliation: Blueprint Medicines, Cambridge, MA 02140, USA

**Keywords:** Pooled shRNA screen, MYC, JAK2, PIM, drug combination, myeloproliferative neoplasms

## Abstract

Inhibitors of JAK2 kinase are emerging as an important treatment modality for myeloproliferative neoplasms (MPN). However, similar to other kinase inhibitors, resistance to JAK2 inhibitors may eventually emerge through a variety of mechanisms. Effective drug combination is one way to enhance therapeutic efficacy and combat resistance against JAK2 inhibitors. To identify potential combination partners for JAK2 compounds in MPN cell lines, we performed pooled shRNA screen targeting 5,000 genes in the presence or absence of JAK2 blockade. One of the top hits identified was MYC, an oncogenic transcription factor that is difficult to inhibit directly, but could be targeted by modulation of upstream regulatory elements such as kinases. We demonstrate herein that PIM kinase inhibitors efficiently suppress MYC protein levels in MPN cell lines. Overexpression of MYC restores the viability of PIM inhibitor-treated cells, revealing causal relationship between MYC down-regulation and cell growth inhibition by PIM compounds. Combination of various PIM inhibitors with a JAK2 inhibitor results in significant synergistic growth inhibition of multiple MPN cancer cell lines and induction of apoptosis. Mechanistic studies revealed strong downregulation of phosphorylated forms of S6 and 4EBP1 by JAK2/PIM inhibitor combination treatment. Finally, such combination was effective in eradicating *in vitro* JAK2 inhibitor-resistant MPN clones, where MYC is consistently up-regulated. These findings demonstrate that simultaneous suppression of JAK2 and PIM kinase activity by small molecule inhibitors is more effective than either agent alone in suppressing MPN cell growth. Our data suggest that JAK2 and PIM combination might warrant further investigation for the treatment of JAK2-driven hematologic malignancies.

## INTRODUCTION

JAK2 is one of important members of Janus kinase family, which mediates cytokine signal transduction to regulate cell proliferation, survival, and differentiation [1]. JAK2 is known to play a significant role in hematopoiesis and immune responses, and is often involved in cytokine dependent cancers. JAK2 fusions have been identified in a variety of blood cancers in which JAK/STAT signaling cascade is constitutively activated [2-5]. In 2005, V617F point mutations in JAK2 were identified in a subset of myeloproliferative neoplasm (MPN) patients. This mutant was later shown to induce MPN like phenotypes in mouse models [6-11]. It is believed that V617F mutation enables JAK2 to be constitutively active by reliving the negative regulatory interaction between its kinase and pseudo-kinase domains.

It has been further demonstrated that JAK2 blockade results in the inhibition of growth of MPN cells harboring JAK2(V617F) mutant [12-14]. As a result, several JAK2 inhibitors have entered clinical trials for hematologic malignancies. Ruxolitinib® (Jakafi) was the first JAK2 inhibitor to be approved by the FDA for the treatment of intermediate and high risk myelofibrosis. While many JAK2 inhibitors are able to achieve normalization of leukocytosis and thrombocytosis, as well as improve symptoms in cancer patients [15, 16], they are less effective in achieving consistent hematologic remissions and reducing JAK2(V617F) allelic burden [15, 17-20].

It is known that JAK-STAT pathway activation in MPN may be caused by mechanisms other than JAK2(V617F) mutation [21]. For example, genetic alterations in the transmembrane domain of MPL can also contribute to JAK-STAT activation and cytokine independent growth [21]. Thus, it is doubtful that JAK2 inhibitors alone would be able to achieve durable responses in all MPN patients. This has prompted further research into more effective therapeutic strategies to combat MPN, specifically combination therapies. Potent combination therapies might not only enhance the efficacy of JAK2 inhibitors, but also limit the unwanted side effects by lowering the dose of JAK2 inhibitors required to achieve the overall therapeutic effect. Importantly, combination therapies have greater chance of preventing early resistance to targeted JAK2 therapy. Although no additional JAK2 mutations have been detected thus far in MPN patients undergoing JAK2 inhibitor treatment, results of several in vitro studies suggest that JAK2 inhibitors may in fact be prone to resistance mediated by novel point mutations in JAK2 itself as well as through activation of other pathways [22-26].

Several combinations with JAK2 inhibitors have been reported recently with beneficial effects on growth inhibition of cells with JAK2 mutations. For example, JAK2 inhibitors work synergistically with HDAC inhibitor panobinostat in inhibiting JAK2 mutant cells [11, 27], and clinical trials have been initiated based on such findings. Similarly, an HSP90 inhibitor enhances the anti-proliferative effects of JAK2 inhibitors by destabilizing JAK2 proteins [28, 29]. Importantly, the latter combination was able to overcome resistance to JAK2 inhibitors in vitro [28, 29]. However, since both HDAC and HSP90 inhibitors have pleiotropic effects, their toxicity in combination with JAK2 suppression might neither be easily predictable nor manageable.

To identify potent combination partners for JAK2 inhibitors, we utilized loss-of-function genomics approach to search for targets that, when knocked down, could synergize with JAK2 inhibition. This was achieved through a pooled shRNA screen in the presence of various concentrations of JAK2 inhibitor SAR302503 (also known as TG101348 [12-14]) or DMSO control. We report here that shRNA-mediated depletion of MYC strongly synergizes with SAR302303 in suppressing viability of MPN cell lines. Since direct pharmacological targeting of MYC remains challenging, we pursued an orthogonal approach to reduce MYC protein levels using pan-PIM kinase inhibitors. As demonstrated herein, JAK2 and PIM inhibitors strongly synergize to inhibit the growth and induce apoptosis in MPN cell lines SET2 and UKE1, as well as in JAK2 inhibitor-resistant clones. Our data suggest that a combination of JAK2 and PIM inhibitors might warrant further investigation for the treatment of JAK2-driven hematologic malignancies.

## RESULTS

### MYC is the top hit in a pooled shRNA library screen to identify combination partners for JAK2 inhibitor

**Figure 1:**
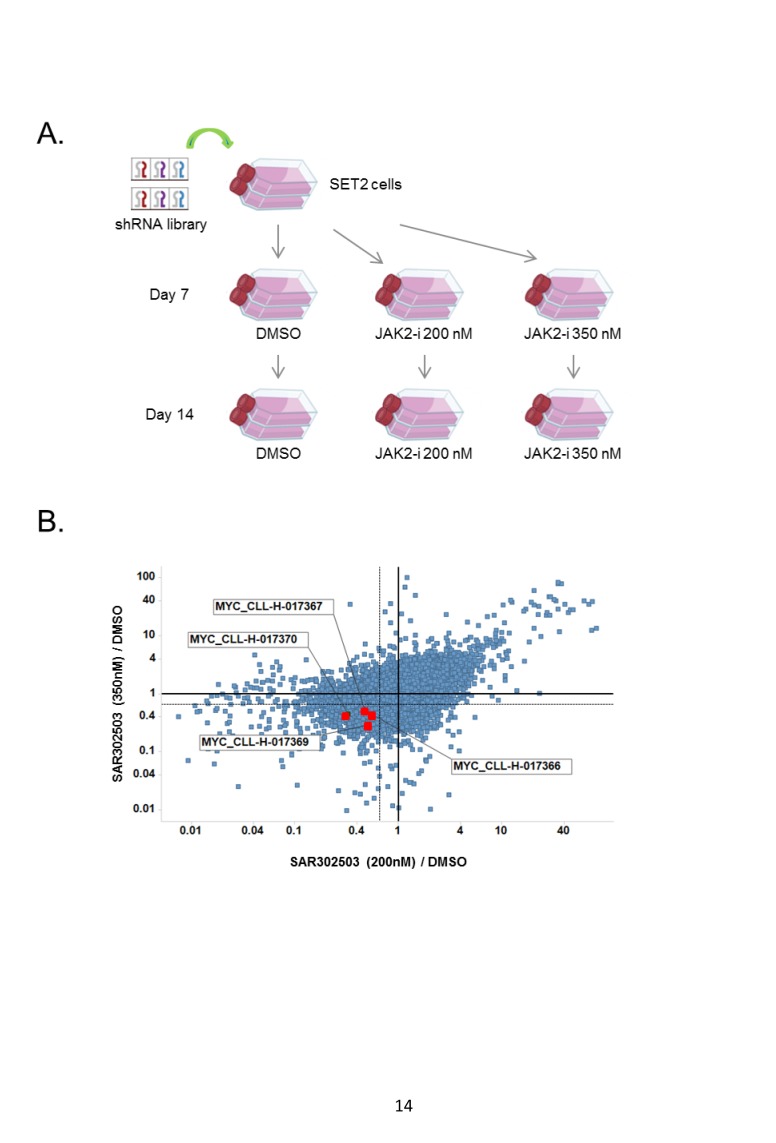
MYC is a top hit in a pooled shRNA screen to identify combination partners for JAK2 inhibitor (A) Schematic representation of pooled shRNA screen performed. SET2 cells were infected with a shRNA library targeting 5,000 genes, and selected with puromycin for 3 days before seeding in 10-cm-plates at a density of 30% for treatment with DMSO control or with JAK2 inhibitor SAR302503 at 0.2 and 0.35 μM. Cells were harvested at 0, 7 and 14 days post drug treatment and cellular genomic DNA was subjected to amplification followed by deep sequencing to determine shRNA copy number. (B) The phenotype of combinational effects was revealed by comparing the quantity of each shRNA in the sample treated with JAK2 inhibitor versus that in the sample treated with DMSO. X-axis: samples treated with 200 nM SAR302503 vs. DMSO; Y-axis: samples treated with 350nM SAR302503 vs. DMSO. 1.5-fold decrease in shRNA abundance is marked with dotted lines. Four MYC shRNAs are marked.

We undertook a systematic shRNA screening approach to identify combination partners for JAK2 inhibitor SAR302503 using SET2 cells, an MPN cancer cell line harboring JAK2 V617F mutation. SET2 cells were infected with a pooled lentviral shRNA library targeting 5,000 disease related genes (Fig. 1A, also see Materials and Methods). Infection was kept at a multiplicity of infection (MOI) of 0.1 in order to deliver approximately one shRNA per cell. In our experience, such low MOI can greatly enhance the quality of the screening results. Following infection, SET2 cells were selected with puromycin for three days and re-plated at low density before initiating treatment with DMSO or JAK2 inhibitor at 0.2 and 0.35 µM, which are the IC25 and IC50 of SAR302503, respectively (Supplementary Fig.1). Cells were harvested at 0, 7 and 14 days post drug treatment and cellular genomic DNA was subjected to amplification and deep sequencing to determine shRNA copy number. Lethal shRNAs, which showed greater than 10 fold decrease in abundance in DMSO treated samples on day 7 or day 14 versus day 0, were excluded from further analysis. The combinational effects were revealed by comparing the quantity of each shRNA in the sample treated with JAK2 inhibitor versus the sample treated with DMSO. To be statistically significant “sensitizer” in this particular screen, each shRNA had to show at least 1.5-fold difference between the treatments, meaning that it sensitized the cells to JAK2 inhibition by at least 1.5-fold. After the shRNAs passing the above criteria were identified, they were further ranked by the number of shRNAs present per gene. MYC was ranked as a top hit with four out of five shRNAs scoring as sensitizers to JAK2 inhibitions at three different concentrations at day 14 (Fig. 1B, supplementary Fig.2). Additional hits with 2-3 shRNAs per gene were shown in Supplementary Fig. 3. To validate MYC as a hit, SET2 cells were stably infected with inducible MYC shRNA. Doxycycline inductions depleted MYC shRNA efficiently, and led to about 2-fold sensitization of SET2 cells to JAK2 inhibitor SAR302503, as measured by cell viability (Supplementary Fig.4). Taken together, our results identify MYC oncogene as potential combination partner for JAK2 inhibitors.

### Inhibition of PIM kinases targets MYC for degradation and recapitulates the effect of MYC shRNA in MPN cells

**Figure 2:**
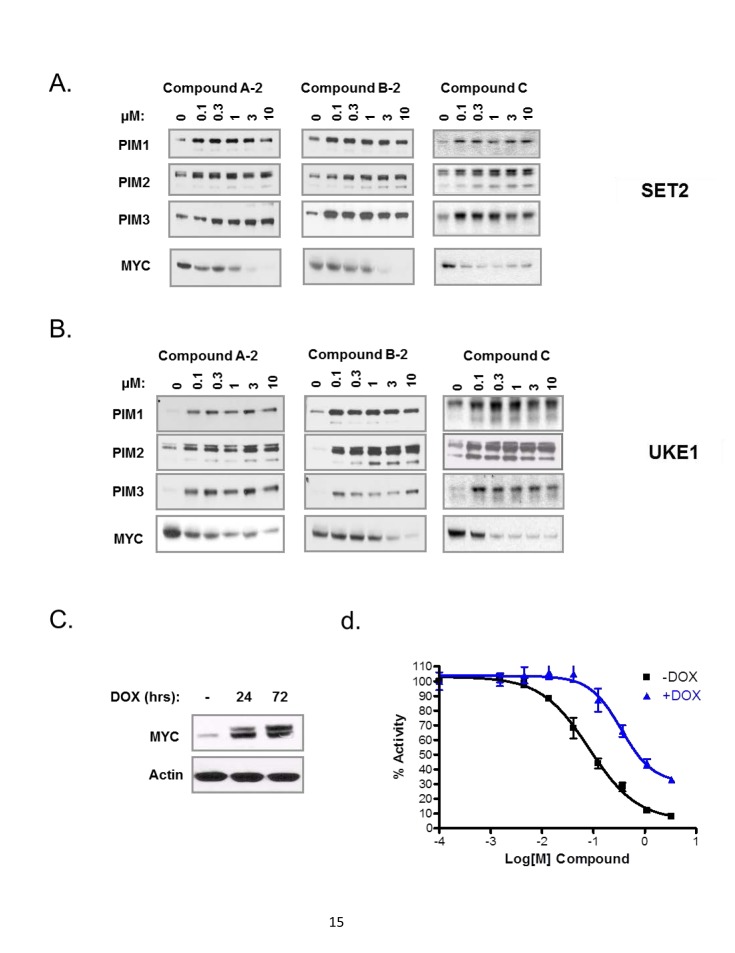
PIM kinase inhibitors downregulate MYC, a critical target of their anti-proliferative effects in MPN cells SET2 (A) and UKE1 cells (B) were treated with three PIM inhibitors for 4 hours and then analyzed by immunoblot using indicated antibodies. (C) UKE1 cells were stably infected with doxycycline-inducible MYC expression vector and MYC protein induction was confirmed by immunoblot analysis after 24 and 72 hour incubation. (D) MYC-UKE1 cells were cultured with or without doxycycline and treated with DMSO or serial dilutions of PIM inhibitor (compound-C). Cell viability measurements were taken 72 hours later.

Since MYC druggability remains a significant challenge, we decided to focus on potential upstream modulators that control MYC protein abundance. One group of such regulators is the PIM family kinases which directly phosphorylate MYC on Ser329 resulting in MYC stabilization [30]. Knock-down of PIM isoforms has been shown to dramatically reduce MYC protein levels [30]. To test our hypothesis that PIM kinase inhibition might de-stabilize MYC protein and thus recapitulate the effect of MYC shRNA, we treated two MPN cell lines, SET2 and UKE1, with three pan-PIM inhibitors representing distinct chemical classes (Supplementary Fig. 5). Strikingly, all three PIM inhibitors significantly reduced MYC protein levels at relatively low compound concentration (Fig 2A, B). In addition, PIM1/2/3 proteins were stabilized in the presence of PIM kinase inhibitors, consistent with the inhibition of PIM autophosphorylation and suppression of negative feedback loop of PIM itself.

MYC is one of several substrates of PIM kinases. We therefore sought to establish that suppression of MYC protein levels by PIM inhibitors is a key determinant of their anti-proliferative activity in MPN cells. To this end, MYC was cloned into a doxycycline-inducible vector and stably transfected into UKE1 cells. UKE1 stable cell line was then treated with increasing concentrations of PIM inhibitor compound C, in the presence or absence of doxycycline. Expression of MYC was confirmed by immunoblot 24 hours and 72 hours after doxycycline induction (Fig. 2C). When MYC was expressed, we observed significant rescue of anti-proliferative phenotype of PIM inhibitors (Fig. 2D). The effect was particularly pronounced at lower concentrations of the PIM compound. These results highlight the critical role of MYC downregulation in the overall anti-proliferative effect of PIM inhibitors.

### JAK2 and PIM inhibitors act synergistically to inhibit proliferation and induce apoptosis in MPN cell lines

**Figure 3:**
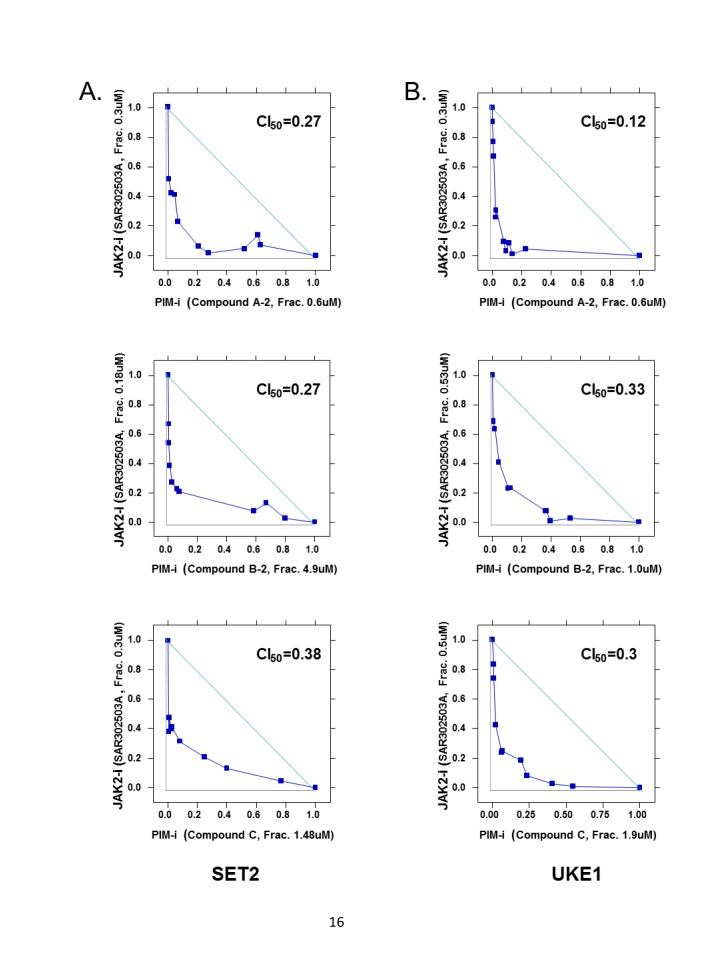
Synergistic effects of JAK2 and PIM inhibitors on MPN cell viability SET2 (A) and UKE1 (B) cells were treated with the combination of JAK2 inhibitor SAR302503 and three PIM inhibitors. Cell viability was assessed 72 hours later. Isobologram analysis was performed as described in Materials and Methods. Combination index (CI) was calculated as the sum of IC50combo/IC50JAK2 and IC50combo/IC50PIM according to Chou-Talay's method. The lowest combination index is shown. Results represent triplicate experiments.

We next asked if, similar to MYC shRNA, PIM inhibitor could indeed be a good combination partner for JAK2 inhibitor. To test this, SET2 and UKE1 cells were treated with a combination of the JAK2 inhibitor SAR302503 and three distinct PIM1/2/3 inhibitors, utilizing nine concentrations for each compound. To formally assess synergy between the two compounds tested, isobologram analysis was performed and a combination index (CI) was computed for each drug combination according to Chou-Talalay's method [31]. Combination index values below 1.0 usually signals synergy, values around 1.0 represent additivity and those above 1.0 represent antagonism between the two drugs tested. All three PIM inhibitors tested herein demonstrated strong synergistic effects with SAR302503, with CI values around 0.2 in both SET2 and UKE1 cells (Fig. 3). Similar, although weaker, synergistic effects were also observed when Ruxolitinib® (JAKafi) was combined with the same PIM inhibitors (data not shown), indicating that PIM and JAK2 inhibitors form a synergistic combination to inhibit the growth of MPN cell lines.

**Figure 4:**
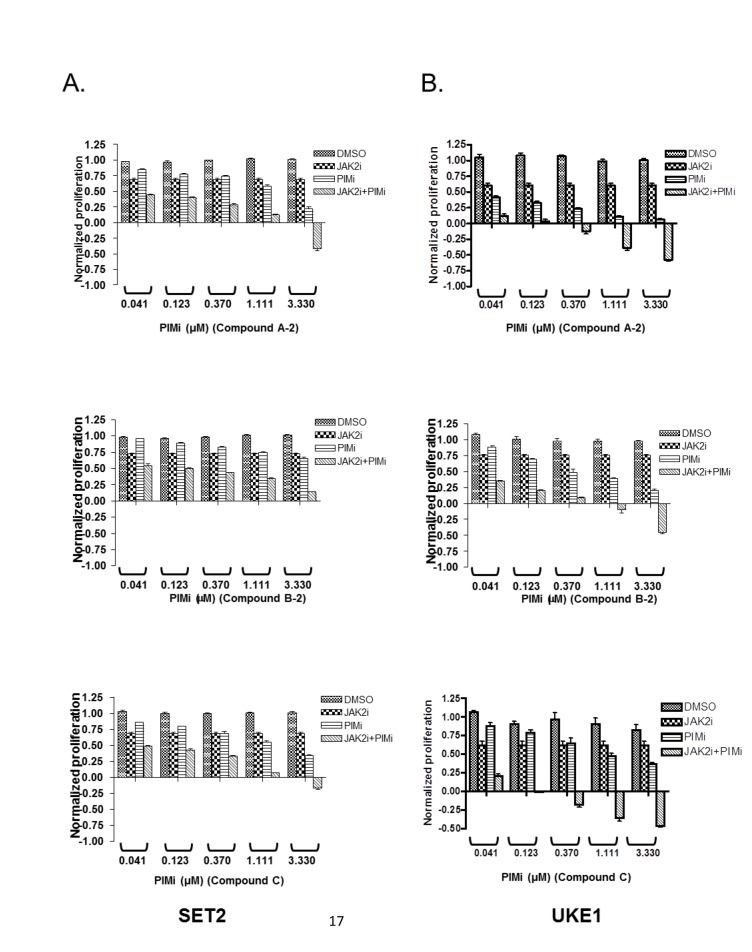
Combination of JAK2 and PIM inhibitors is more efficacious than either agent alone in suppressing MPN cell viability SET2 (A) and UKE1 (B) cells were exposed to the constant amount of JAK2 inhibitor (0.3 μM for SET2 and 1 μM for UKE1), while PIM compounds were titrated in a dose response format. Cell viability was measured using CellTiter-Glo® kit before (CTGbefore) and after 3 days of drug treatment (CTGafter). Effects of drug treatments were calculated as a ratio of (CTGafter-CTGbefore)/ CTGbefore. CTGafter=CTGbefore indicates cell stasis; CTGafter<CTGbefore indicates active cell death. Experiments were performed in triplicate.

To confirm and extend these observations, we repeated drug combination experiments, now holding the concentration of JAK2 compound constant and below the IC50 value, while titrating in PIM inhibitors at five different concentrations. As can be seen in figure 4, JAK2 inhibitor alone inhibited proliferation of SET2 and UKE1 cells by approximately 25% at the concentration tested. PIM inhibitors caused dose-dependent reduction in cell viability, which was significantly potentiated by the addition of the JAK2 inhibitor. Most importantly, JAK2/PIM combination appeared to have reduced the cell number below initial plating density implying that active cell death, rather than inhibition of cell proliferation, was taking place. This phenotype was more pronounced in UKE1 cells (Fig. 4B).

**Figure 5:**
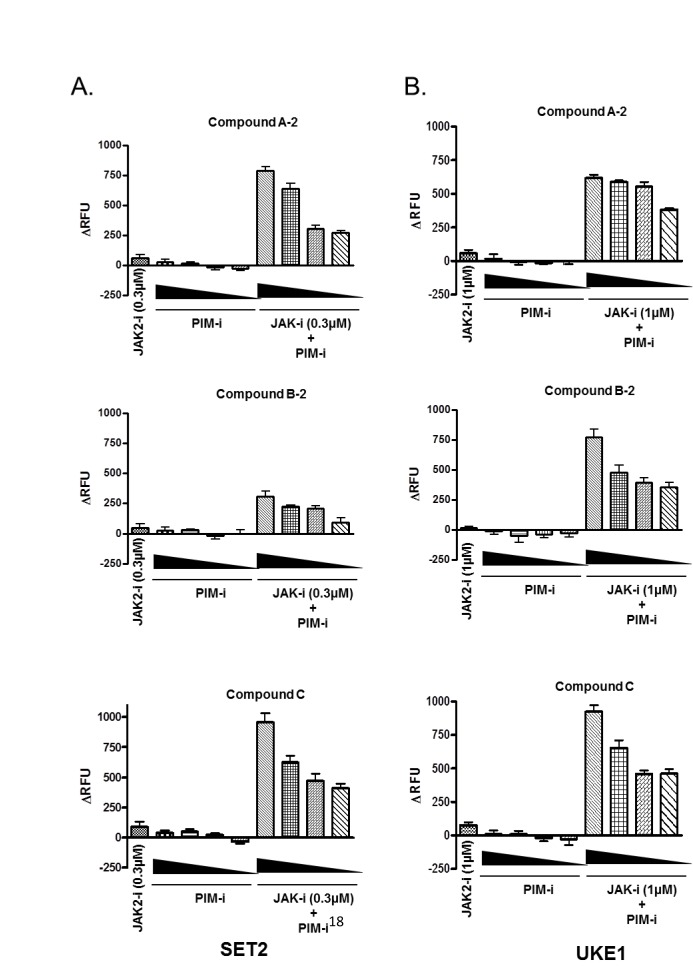
JAK2 and PIM inhibitor combination induces apoptosis in MPN cells SET2 (A) and UKE1 (B) cells were exposed to the constant amount of JAK2 inhibitor (0.3 μM for SET2 and 1 μM for UKE1), while PIM compounds were titrated in a dose response format. Apoptosis induction was determined after 3 days of treatment by using Homogeneous Caspase Assay kit as described in Materials and Methods. Results of triplicate experiments are shown.

To further investigate the impact of combined JAK2/PIM blockade on the induction of apoptosis in MPN cell lines, SET2 and UKE1 cells were treated with a fixed concentration of JAK2 inhibitor and an increasing amount of each of the three PIM inhibitors. Caspase activity was used as a read-out for apoptosis induction. As shown in figure 5, neither JAK2 nor PIM inhibitor alone induced apoptosis at the doses tested. However, apoptosis was greatly increased in both cell lines and in a dose dependent manner when JAK2 compound was combined with either PIM inhibitor (Fig. 5). Taken together with cell viability data, these results strongly suggest that JAK2 and pan-PIM inhibitors act synergistically to induce caspase activation and cell death in MPN cell lines.

### JAK2/PIM inhibitor combination suppresses pro-survival signaling in MPN cell lines

**Figure 6:**
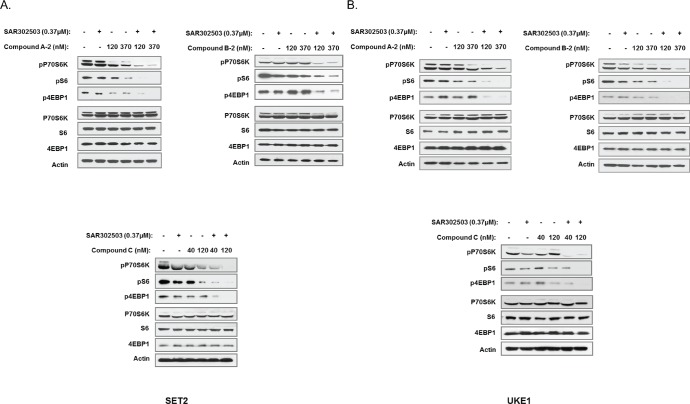
Simultaneous inhibition of JAK2 and PIM affects critical signaling nodes in MPN cells SET2 (A) and UKE1 (B) cells were treated with JAK2 and PIM inhibitors for 4 hours, either alone or in combination, at indicated doses. Immunoblot was used to analyze total and phosphorylated forms of 4EBP1, S6 and P70S6K in treated cells.

JAK/STAT signaling is known to depend on PIM expression to exert its pro-survival effects in certain cell types [32]. In turn, PIM controls cellular survival and proliferation through a plethora of downstream targets, including transcription factors (MYC), components of protein translation machinery (4EBP1), regulators of apoptosis (BAD) and cell cycle (CDC25A/C) and many others [33-35]. Therefore, in order to obtain additional mechanistic insights into synergistic activity of JAK2 and PIM inhibitors, we examined key biochemical readouts that are known to be regulated by JAK/STAT/PIM signaling. Phosphorylation of pro-survival P70 S6 kinase (pP70S6K), ribosomal protein S6 (pS6) and a key mediator of protein translation 4-EBP1 (p4EBP1) were significantly reduced by the combination of JAK2 and PIM inhibitors in both SET2 and UKE1 cells, while each agent alone exerted only modest effects (Fig. 6). Importantly, all three PIM inhibitors displayed similar synergistic activity with the JAK2 inhibitor on downstream signaling, suggesting that the observed effects are not compound-specific. These results solidify our earlier phenotypic observations and offer mechanistic basis for strong combinatorial effects of JAK2 and PIM inhibitors in suppressing MPN cell growth.

### JAK2 inhibitor-resistant clones are sensitive to JAK2/PIM drug combination

**Figure 7:**
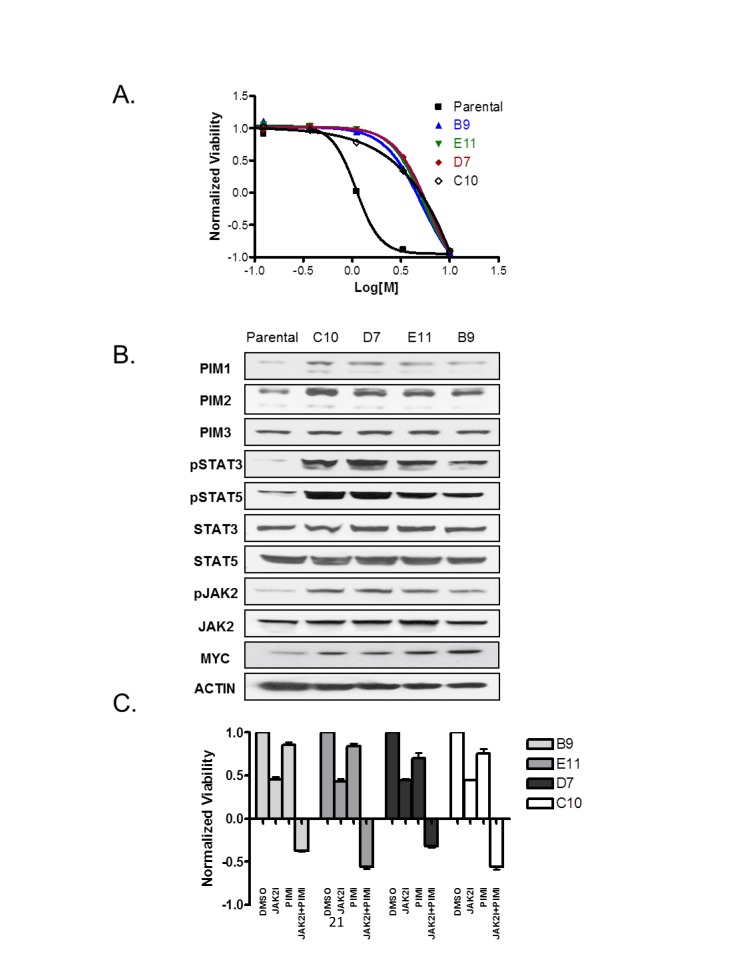
Combination of JAK2 and PIM inhibitors is effective against JAK2 inhibitor-resistant cells (A) SET2 cells were exposed to 2 µM of JAK2 inhibitor for 8 weeks to generate resistant clones. Cell viability of four resulting clones, as well as parental cells, was subsequently evaluated in the presence of a dose response of JAK2 compound. (B) Resistant clones were analyzed by immunoblot using indicated antibodies to detect changes in critical survival nodes. (C) Resistant cells were treated with 3 µM of either JAK2 or PIM inhibitor (compound C) alone or in combination for 3 days. Cell viability was measured using CellTiter-Glo® kit before (CTGbefore) and after 3 days of drug treatment (CTGafter). Effects of drug treatments were calculated as a ratio of (CTGafter-CTGbefore)/ CTGbefore. CTGafter=CTGbefore indicates cell stasis; CTGafter<CTGbefore indicates active cell death. Triplicate experiments are shown.

Rapidly emerging resistance to targeted kinase inhibitors is of great concern and might eventually undermine clinical utility of these agents in the treatment of cancer patients. Therefore, effective drug combinations are urgently needed to combat such resistance. We set out to generate JAK2 inhibitor-resistant clones and test JAK2/PIM drug combination in this setting. To induce resistance, SET2 cells were continuously cultured in the presence of IC90 of JAK2 inhibitor (2 µM) for 8 weeks. Four individual clones were isolated and characterized for sensitivity to JAK2 compound. As demonstrated in figure 7A, resistant clones were significantly less sensitive to JAK2 inhibitor than parental SET2 cells. All resistant clones consistently exhibited upregulation of MYC, PIM1 and PIM2, while maintaining similar protein levels of PIM3. In addition, phospho-STAT3/5 and phospho-JAK2 were significantly increased in JAK2 inhibitor resistant cells (Fig. 7B). Survival of all four resistant clones was substantially reduced by the combination of JAK2 and PIM inhibitors. Furthermore, we again detected a reduction in relative cell number below the initial plating density, suggestive of cell killing rather than suppression of cell proliferation (Fig. 7C). Our data imply that JAK2/PIM inhibitor combination could be beneficial in the context of resistance to JAK2 monotherapy.

## DISCUSSION

In this study we set out to identify potential combination partners for JAK2 inhibitors in MPN cell lines. Utilizing unbiased screening approach, we discovered that MYC knock-down enhances anti-proliferative effects of JAK2 compound, with four out of five MYC shRNAs meeting the “sensitizer” criteria. It is worthy to note that multiple other hits are known to be involved in MYC network, such as Notch1, Arrb2, Mlh1, Mvd, Nfyc, Tgif2 and others [36]. To circumvent significant druggability challenges associated with direct targeting of MYC, we turned to PIM kinase inhibitors to downregulate MYC protein levels in MPN cells. Incidentally, deep biological connections between MYC and PIM have already been well established. For example, tumorigenesis in transgenic mice co-expressing both Eμ–Pim1 and Eμ–Myc is vastly accelerated, thus highlighting strong synergism between these two oncogenes [37]. In addition, PIM1/2 is able to phosphorylate MYC and dramatically increases MYC protein stability, thereby promoting oncogenic transcriptional activity of MYC [38]. On the other hand, MYC has been shown to upregulate PIM1 in certain tumors [39], further reinforcing the intricate interplay between these two proteins. It should be noted however, other pharmacological agents are capable of downregulating MYC. For example, CDK/CK1 inhibitors have been shown to suppress MYC levels in human neuroblastoma cell lines [40]. It remains to be determined if these inhibitors would behave similarly in MPN cells.

JAK2/PIM inhibitor combination described herein is likely to be one of several strategies to enhance therapeutic efficacy of JAK2 compounds. For instance, combination of JAK1/2 and pan-histone deacetylase (HDAC) inhibitors has already been shown to be well-tolerated in mouse models of JAK2V617F-driven disease and resulted in improved efficacy compared with single agents [27]. Signaling nodes along the JAK2 signaling pathway have also been interrogated for potential combination partners [1, 41]. Co-treatment with mTOR and JAK2 inhibitors had synergistic activity against the proliferation of JAK2V617F mutated cell lines [42]. In another study, a series of serine/threonine kinase inhibitors were tested in combination with JAK2 compounds in BaF3 TpoR JAK2 V617F cells. The strongest synergy was observed with the pan PI3K inhibitors GDC0940 and dual PI3K/mTOR inhibitor BEZ235 [43]. Thus both HDAC and PI3K/mTOR inhibitors may represent examples of additional combination partners for JAK2 compounds. However, our data convincingly show that JAK/PIM combination not only enhances the efficacy of JAK2 inhibitors in “naïve” MPN cells, but can also eradicates MPN cells that have become resistant to JAK2 monotherapy The increased MYC protein levels observed in the resistant cells described in this work might be the underlying or a contributing resistance mechanism, and MYC destabilization via PIM inhibition a means to resensitize these cells to JAK2 inhibition. In addition, the safety profile of pan-PIM inhibitors is likely to be more favorable than other combination partners since PIM1/2/3 knock-out mice are viable and fertile [44].

Our data suggest that JAK2/PIM, as well as other JAK2 combinations, might be converging on essential signaling nodes in MPN cells, including pP70S6K and p4EBP1, both of which are mTORC1 substrates [45]. 4EBP1 is essential for cap-dependent mRNA translation [46], and was recently identified as a druggable target in tumors with MYC activation [47]. Downregulation of p4EBP1 was observed when JAK2(V617F)-expressing cells were treated with the combination of JAK2 inhibitor and pan-PI3K/mTOR inhibitor BEZ235 [28]. Thus, downregulation of 4EBP1 function might represent an important determinant of efficacy of JAK2/PIM combination therapy.

In conclusion, our findings demonstrate that simultaneous suppression of JAK2 and PIM kinase activity by small molecule inhibitors is more effective than either agent alone in inhibiting the growth of MPN cells. This combination is also effective in eradicating JAK2 inhibitor-resistant MPN clones in vitro. If translated into clinical practice, combined JAK2/PIM blockade may be able to enhance the efficacy of current JAK2 inhibitors and combat the threat of emerging resistance.

## MATERIALS AND METHODS

### Cell lines and culture conditions

SET2 cell line was purchased from DSMZ (Braunschweig, GERMANY). UKE1 cell line was a kind gift from Dr. Ross Levine at MSKCC (New York, USA). SET2 cells were cultured in RPMI1640 medium (Gibco, Grand Island, NY, USA) supplemented with 20% fetal bovine serum (FBS, Gibco). UKE1 cells were cultured in IMEM (Gibco) medium supplemented with 10% FBS. Both cell lines were kept in a 37°C humidified incubator containing 5% CO2.

### Compounds

Structures of the compounds used in this study are shown in Supplementary Figure 5. Compound A-2 and Compound B-2 are PIM inhibitors identified from Sanofi internal drug discovery efforts. These compounds inhibit all three PIM isoforms, PIM1, PIM2 and PIM3 with biochemical IC50 ranging from 0.1-30nM. Compound C is a comparably potent PIM inhibitor developed by Novartis [48]. JAK2 inhibitor SAR302503 is also known as TG101348 that inhibits JAK2 with an IC50 of 6nM. All compounds were internally synthesized and stock solutions were diluted in dimethylsulfoxide. (DMSO, Sigma-Aldrich St. Louis, MO, USA)

### Immunoblot

SET2 or UKE1 cells were treated with either single inhibitor or a combination of PIM inhibitor and JAK2 inhibitor respectively for 4 hours at indicated concentrations. The compounds were then washed off by PBS. The cell pellets were collected and lysed with RIPA buffer (Santa Cruz Biotechnology, Dallas, Texas USA) supplemented with HaltTM phosphatase, protease inhibitor cocktail (Thermo Scientific, Rockford, IL USA,) and phenylmethanesulfonyl fluoride solution (PMSF, Sigma-Aldrich). Lysates were resolved on a 4-12% Bis-Tris gel (Life Technologies, Grand Island, NY, USA) and transferred to nitrocellulose membranes. The membranes were blocked for 1 hour at room temperature with 5% milk, then incubated with the indicated primary antibodies at 1:1000 dilution at 4°C overnight. All primary antibodies were purchased from Cell Signaling (Danvers, MA, USA): Pim1 (2907), Pim2 (4730), Pim3 (4165), c-Myc (5605), pS6-Ser240/244 (2215), S6 (2217), p4EBP1-Thr34/46 (2855), 4EBP1 (9644), pP70S6K-Thr389 (9234), P70S6K (2708), β-actin (5125). After washing, the membranes were incubated with secondary HRP-conjugated anti-rabbit antibody (Santa Cruz) at 1:3,000 for 1 hour at room temperature.

### Cell viability/proliferation assay and synergism assessment

Log growth phase cells were suspended in growth medium and seeded into 96-well plates at density of 1.6×104 per well and treated with either single inhibitor or combinations of PIM inhibitor and JAK2 inhibitor respectively for 72 hours at 37°C. Serially diluted compounds (up to 10μM) were used in all studies. Cell viability/proliferative expansion was assessed by addition CellTiter-Glo® reagent (Promega, Madison, Wisconsin, USA, G7573) for 10 minutes, and endpoint readings were collected using a SpectraMax Pro V5 plate reader (Molecular Devices, Sunnyvale, CA, USA). The intensity of the luminescence signal was directly proportional to the number of live cells. The IC50 of single compound was determined with XLfit and Biost@t-SPEED. The combination index (CI) was used to evaluate the interaction between PIM inhibitor and JAK2 inhibitor. Synergy was described using an isobologram, which compared the doses needed to reach 50% inhibition (IC50, calculated with Xfit) along an equal-effect contour to those along a predicted straight line based on a model of dose additivity effect. Combination index was calculated using the formula CI = CX/IC50X + CY/IC50Y, where CX or CY is the concentration of compound X or Y in a 50% effective mixture of the most potent combination, and IC50X or IC50Y is the IC50 of either compound when applied as single agent. CI values of <1 indicate synergism, CI =1 indicate additivity and CI>1 indicate antagonism.

### Apoptosis/Caspase activity assay

Apoptosis/caspase activity was measured in actively growing SET2 and UKE1 cells seeded into 96-well plates at density of 3.2×104 per well and treated with either single inhibitor or combinations of PIM inhibitor and JAK2 inhibitor at indicated concentrations for 24 hours at 37°C. Homogeneous Caspase Assay (Roche Applied Science, Indianapolis, USA) was used according to vendor instructions.

### Inducible UKE1-MYC cell line

Trans-Lentiviral ORF Packaging Kit (Thermo Scientific, TLP5918) was used according to manufacturer's instructions to prepare MYC-expressing lentivirus. Briefly, packaging plasmids and MYC expression plasmid or an empty vector were transiently co-tranfected into HEK293T cells and lentiviral supernatants were collected and concentrated using Lenti-X concentrator (Clontech, Mountain View, CA USA). UKE1 cells were infected with lentivirus in the presence of 8μg/ml polybrene (Millipore, Billerica, MA, USA) with the concentrated lentiviruses at different MOI. The transduced cells were cultured in selection medium including 1μg/ml puromycin.

### JAK2 inhibitor-resistant SET2 cell lines

SET2 cells were continuously cultured in the growth medium containing 2 µM of SAR302503 for 8 weeks to generate resistant clones. Four individual clones (B9, C10, D7, E11) were chosen for further analysis and routinely maintained in the culture medium containing 2 µM of SAR302503.

## SUPPLEMENTARY FIGURES


