# Supplementary material for: Combination of PIM and JAK2 inhibitors synergistically suppresses cell proliferation and overcomes drug resistance of myeloproliferative neoplasms
Source: Oncotarget. 2014 May 8;5(10):3362–74. doi: 10.18632/oncotarget.1951 (PMC4102815; doi:10.18632/oncotarget.1951)
Supplement: Supplementary file 1 [file oncotarget-05-3362-s001.docx]

**Combination of PIM and JAK2 inhibitors synergistically suppresses cell proliferation and overcomes drug resistance of myeloproliferative neoplasms**

Supplementary Figure 1: Selection of compound concentrations for SET2 treatment in the pooled shRNA screen


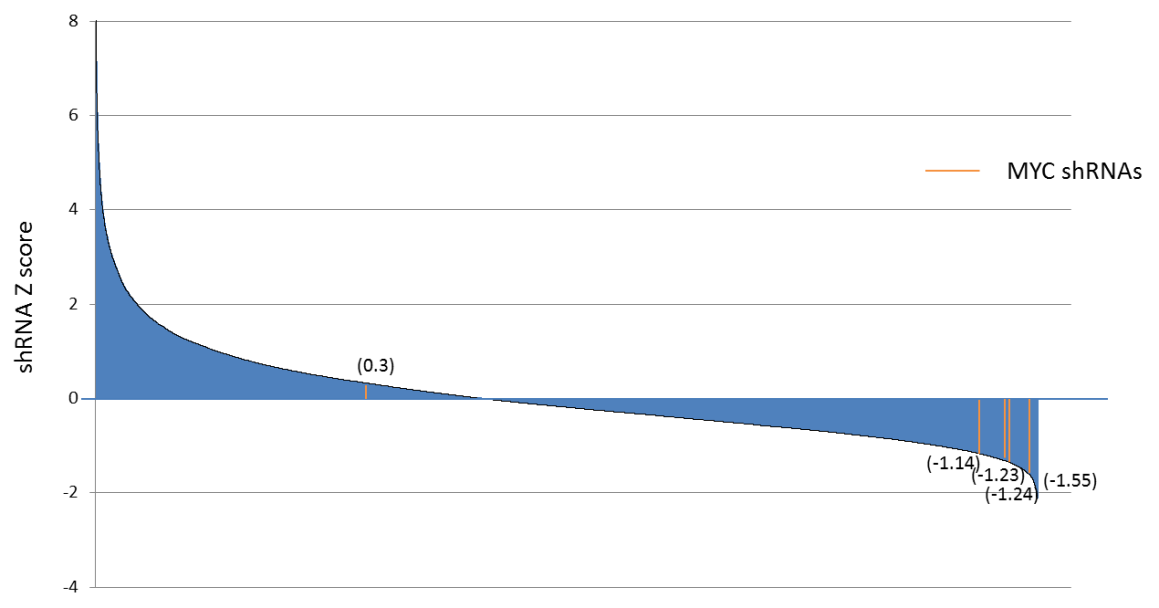


Supplementary Figure 2: Z scores of five MYC shRNAs in the pooled shRNA screen for JAK2 inhibitor sensitizers


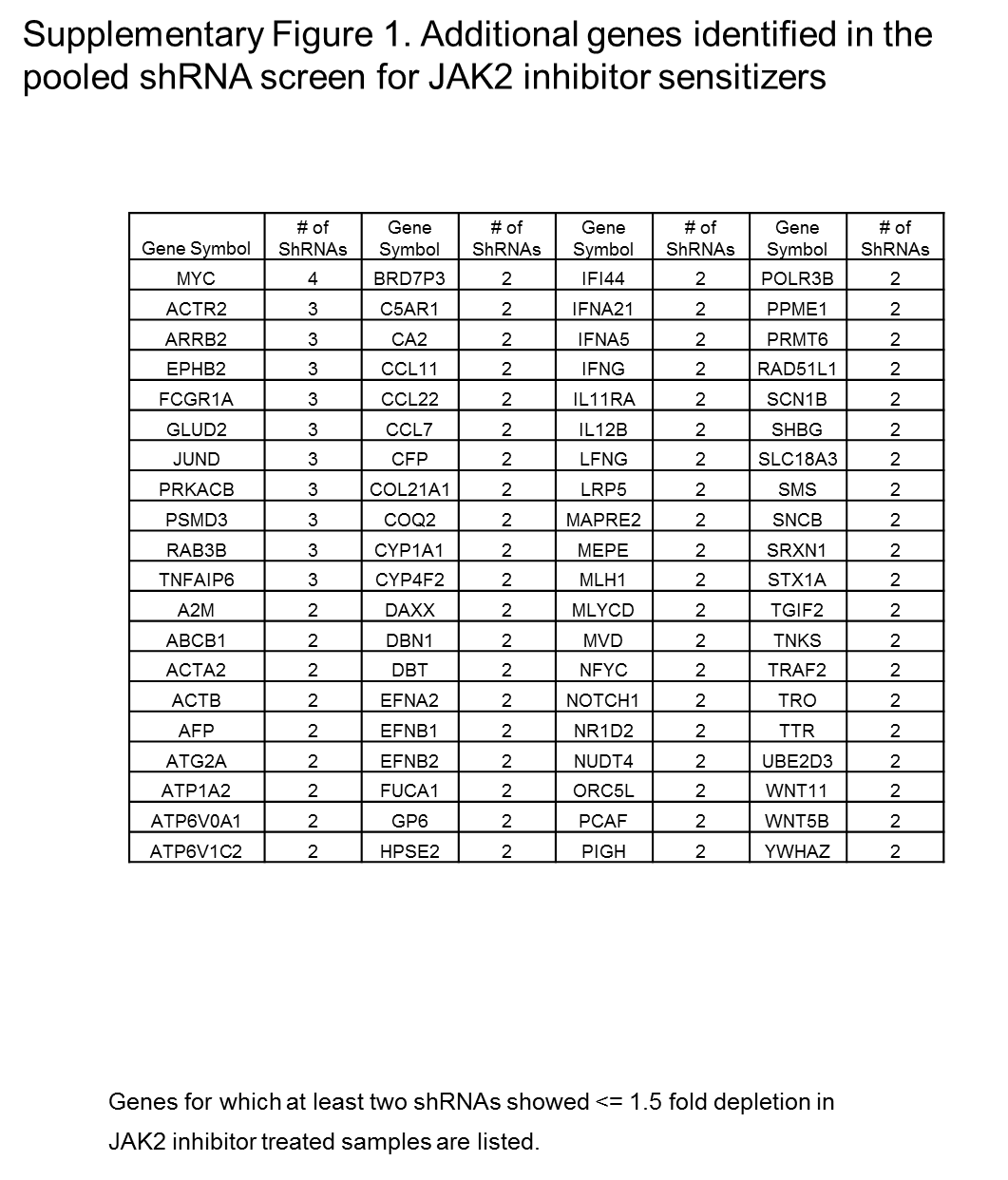


Supplementary Figure 3: Additional genes identified in the pooled shRNA screen for JAK2 inhibitor sensitizers

A

B

Supplementary Figure 4: Knock-down of MYC by shRNA sensitizes SET2 cells to JAK2 inhibition

Supplementary Figure 5: Chemical structures of PIM inhibitors
